# Key factors advancing food education in primary schools – Perspectives of headteachers and education directors

**DOI:** 10.1017/S1368980025101547

**Published:** 2025-12-10

**Authors:** Aija L. Laitinen, Tanja Tilles-Tirkkonen, Leila Karhunen, Sanna Talvia

**Affiliations:** 1 Department of Public Health and Clinical Nutrition, University of Eastern Finland, Kuopio, Finland; 2 Department of Education, University of Helsinki, Helsinki, Finland

**Keywords:** School headteachers, Nutrition education, Municipal officials, Finnish education system, Qualitative research, Educational policy

## Abstract

**Objective:**

Schools are key environments for promoting healthy eating habits, food knowledge and skills, but the systematic implementation of food education is usually lacking. This study aimed to examine the perceptions of primary school headteachers and municipal education directors regarding the key factors influencing the implementation of food education in Finnish primary schools.

**Design:**

In this qualitative study, the participants took part in research interviews. The interviews were transcribed verbatim and analysed using theory-driven content analysis to identify common categories.

**Setting:**

Semi-structured one-on-one interviews were conducted.

**Participants:**

The interviews involved twelve headteachers and five education directors, all of whom had prior experience in implementing food education through the Tasty School project, which supported primary schools in delivering food education.

**Results:**

In the analysis, the key factors influencing implementation of food education were categorised according to an ecological framework into two levels: the macro level and the school community level, which represents the micro-level interactions within the school’s physical and social environment. The results indicate that successful food education requires a school culture that prioritises it − incorporating curriculum integration, dedicating adequate planning time and ensuring sufficient resources.

**Conclusions:**

Primary schools would benefit from a school culture that prioritises food education. This includes setting objectives in the curriculum, allocating sufficient time for planning, ensuring resources and creating supportive learning environments. While headteachers play a central role, support from municipal officials is essential for sustained implementation. These findings provide insights to support the implementation of food education at both school and municipal levels.

## Graphical abstract

**Figure:**
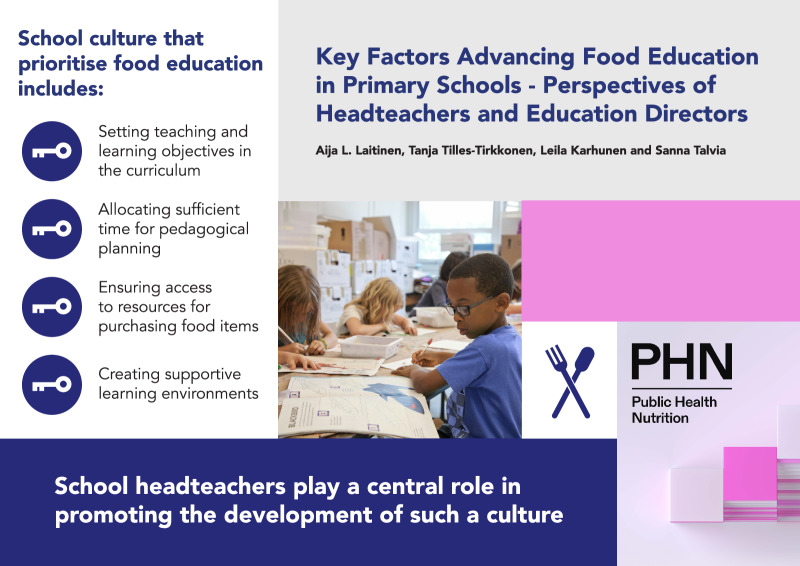

Multiple studies have shown that poor dietary behaviour, such as irregular breakfast consumption, negatively impacts academic performance^(1–3)^. Childhood and adolescence are critical periods of physical, mental and cognitive development, all of which are closely linked to nutritional status^(4,5)^. Schools are seen as critical venues for reaching children and effectively promoting knowledge and skills of food and nutrition, as well as the social culture surrounding healthy eating habits^(6–8)^. However, the implementation of food education in schools is not carried out systematically, and the International Food Policy Research Institute’s 2016 Global Food Policy Report has subsequently called for research into the challenges and facilitators of food education implementation in schools^(9)^. Developing an understanding among policymakers, school staff and academics regarding the challenges and opportunities of implementing food education is crucial to fully capitalise on the potential benefits it offers to school communities and public health.

In Finland, food education in schools is guided by the Basic Education Act, the National Core Curriculum for Basic Education^(10)^, the National School Meal Recommendation^(11)^ and the municipalities’ local curricula. According to the Basic Education Act, every child participating in basic education must be provided, on each school day, with a free, well-balanced meal that is appropriately organised and supervised (Finnish Law: Basic Education Act 628/1998). The national core curriculum directs teaching and educational activities in primary schools and recognises school meals as part of both school operations and food education^(10)^. However, it does not define specific objectives or content for food education, nor does it describe accurately the methods of implementation. In the subject-specific sections, food and nutrition are most often mentioned in connection with Home Economics and Health Education, but these subjects play only a minor role in primary school. Health Education is integrated with several other subjects into Environmental Studies, and Home Economics is an optional subject that is not offered in all primary schools. In primary education, classroom teachers are responsible for teaching several subjects, which, on the other hand, enables the integration of food education into various subjects, supporting multidisciplinary and phenomenon-based learning. However, food education is not included in class teacher training; thus, its implementation relies mainly on teachers’ personal interest in the topic and their inherent understanding of it^(12)^. In conclusion, food education is not a separate subject in Finnish primary schools; rather, it is intended to be linked to the objectives of transversal competencies and integrated into subjects and universal school dining^(10)^.

Possible objectives for food education, as identified in previous research, aim to support healthy and sustainable eating habits and promote a positive relationship with food among children and adolescents, as well as to enhance their comprehensive understanding of food-related phenomena^(13–16)^. Several interventions, both in Finland and globally, have shown that school-based food education initiatives promote children’s eating patterns and could thus help prevent lifestyle-related diseases^(6,17,18)^. However, if food education practices are primarily addressed through short-term interventions introduced from outside the school, they may fail to reach broader populations, and the investments in time, personnel and resources required to initiate and implement these practices may be ineffective^(19)^. Therefore, the knowledge, competencies and perceptions of school stakeholders and staff regarding the overall implementation of food education in their teaching practices must be considered.

Globally, teachers perceived that a lack of time has been identified as a key challenge to implementing food education in compulsory education^(20–25)^. This lack of time is often related to broader curricular priorities and the marginalisation of food education as a distinct area of learning within the school curriculum. Notably, such time-related challenges experienced by teachers have been shown to decrease as they gain experience in implementing food education^(26)^. In addition to time pressures, the literature highlights a range of other challenges. Heavy workloads assigned to teachers^(26,27)^, limited financial resources in schools^(20,23,25,28)^, inadequate curricula and teaching materials^(7,24,28,29)^, insufficient knowledge and skills among school staff^(20,27,29)^, a lack of professional development opportunities^(27,28,30)^, insufficient support from school leadership^(24,27,30)^ and the physical environment of the school^(20,23,25)^ have also been perceived as challenges to the implementation of food education.

Previous studies have also identified factors that facilitate the implementation of food education. Teachers generally have a positive attitude towards food education and consider supporting pupils’ health and wellbeing to be important^(7,23,24)^. School leadership’s commitment and support are considered crucial for the successful implementation of a whole-school approach to food education^(7,23)^. According to teachers’ own experiences, leadership’s commitment to the issue makes it more likely that they will incorporate food education into their teaching^(28)^. Ready-made food education materials and lesson plans reduce teachers’ workload and thus increase their willingness to implement food education^(24–26)^. Specific food education models have also been found to be feasible for schools, as they provide valuable resources for teachers and generate interest in the practical implementation of food education^(23,31)^.

Research on the delivery of food education in school settings has so far focused primarily on teachers. Consequently, the factors identified as challenges and facilitators of food education implementation largely reflect the teachers’ perspective, with many highlighting the critical role of leadership support in successful implementation. However, a wide range of professionals contribute to food education, including headteachers and municipal education directors, who are responsible for setting educational policies and allocating resources. Therefore, it is essential to gather research-based information on the factors influencing food education implementation from the perspectives of these key stakeholders as well^(7,23,25)^.

This study aimed to examine the perceptions of primary school headteachers and municipal education directors regarding the key factors influencing the implementation of food education in Finnish primary schools. The findings provide insights into opportunities for actions that strengthen food education implementation.

## Methods

In this qualitative research study, we conducted seventeen semi-structured, one-on-one interviews in the Finnish language with education directors (*n* 5) in February 2019 and with primary school headteachers (*n* 12) between February and April 2020 (Table 1). The participants worked in six different municipalities across Eastern and Southern Finland, with populations ranging approximately from 10 000 to 230 000. The primary schools led by the headteachers varied in size, ranging approximately from 40 to 380 pupils, with an average enrolment of 175 pupils, representing schools of typical size in Finland. To ensure that the participants were familiar with food education in schools, all interviewees had previously participated in the Tasty School project^(23)^, either in their role as leaders of the intervention schools or by approving the participation of their subordinates in the project. The Tasty School project was a research-based intervention implemented in Finland. As part of the intervention, primary schools in the intervention group were provided with materials and support to implement food education, while control schools did not receive these resources. The interviewees were invited to participate in the research interview via email, and three headteachers and one education director of those invited declined to participate in the study. To ensure reliability, all interviews were conducted by the same researcher (the first author, female, white, MHSc, Registered Dietitian, doctoral researcher in clinical nutrition). The research interviews were conducted in Finnish either in the interviewee’s office (*n* 15) or via the remote video platform *Microsoft Teams* (*n* 2). All participants reviewed the consent form and gave permission for audio recording. Written consent was obtained prior to the start of each interview. The participants were not given the opportunity to review their transcripts. At the beginning, a brief survey was administered to collect demographic data (municipality, position, years in education sector, gender and age). The research team had no dependable relationship with any of the leaders recruited within this study, and the participants did not receive any incentive payment for their participation. The Consolidated Criteria for Reporting Qualitative Research (COREQ) were followed (online Supplementary Material 1).

**Table 1.** Description of participants (*n* 17)

Demographics	*n*	%	Mean	sd	Range
**Position**					
Education director in a municipality	5	29			
Primary school headteacher	12	71			
Years in education sector			22·9	14·0–31·9	7–41
Age in years			48·4	39·5–57·3	31–63
**Gender**					
Female	9	53			
Male	8	47			

In Finland, the roles of headteacher and education director may vary slightly depending on the municipality. The headteacher’s main task is to manage and develop the school’s operations. They are responsible for ensuring that the school’s pedagogical activities, learning environments and practices support the implementation of the curriculum, as well as the wellbeing and learning of the pupils. In addition, the headteacher oversees the school’s finances, personnel management and collaboration with various stakeholders, such as parents and other partners. The municipal education director oversees the administration and development of education within the municipality. Their role is to ensure that education is provided in accordance with the law, national curricula and local objectives. They are also responsible for monitoring the quality of education, managing resources and finances and collaborating with various stakeholders such as schools, headteachers, parents and other municipal departments. In the Finnish primary school system, class teachers are generally responsible for teaching most subjects to their pupils, though some subjects, such as foreign languages, may be taught by subject teacher^(10)^. It is noteworthy that, in Finland, schools have been mandated by law to provide all pupils with a free hot meal since 1948^(32)^. However, school meals are not free for teachers and other school staff.

All authors contributed to the design of a semi-structured interview guide which was designed based on the research purpose. The interview guide was not piloted, as the target group was limited in size, and maximising participation in the study was prioritised. The interview guide had five main sections with open-ended questions and numerical rating tasks (Table 2). In addition to these main interview questions, the respondents were asked probing questions for further clarity of their answers. Field notes were made after the interviews.

**Table 2.** Interview guide

Section	Questions
1) Understanding of food and nutrition education in primary schools	How do you think about what food and nutrition education in primary schools is? Do you think there is a difference in meaning between the concepts of food education and nutrition education? A shared understanding of food education was discussed and explored. Is food education, as described above, generally implemented in primary schools? In what way?
2) Attitudes towards food education	What is the importance of food education in primary schools? Please rate it on a scale of 1–10, with 1 being very unimportant and 10 being very important. Why did you answer (the corresponding number)? What are the benefits of food education in primary schools? What are the disadvantages of food education in primary school? What is the connotation of the word food education? Rate it on a scale of 1–10, with 1 being very negative and 10 being very positive. Why did you answer (the corresponding number)?
3) Barriers and facilitators of food education	What is the implementation of food education like in practice? Please rate on a scale of 1–10, where 1 is very difficult and 10 is very easy. Why is it easy/difficult to implement food education in practice? (the wording of the question depends on the answer to the previous question) What factors make it possible to implement food education in primary schools? What factors make it difficult to implement food education in primary schools?
4) Impact of own activities on food education in primary schools	Can you yourself influence the factors mentioned above? Why/How? Who is responsible for the implementation of food education in primary schools? If food education were now more strongly introduced in primary schools, how would it be received? How do you see yourself as a food educator?
5) Visibility of food education in municipal documents	What kind of knowledge management tools are used at municipal level in relation to children’s eating and food? Would you like to have some other knowledge management tools to support your work on children’s eating and food? Have the eating habits of schoolchildren been examined in the municipal wellbeing report? Is the implementation of food education and its objectives included in the municipal curriculum? Is it reflected wider than just concerning school meals? Is food education visible in school curricula? (asked only from headteachers) Is food education visible in the documents of the food services?

During the first section of the interview, the participants were asked to share their conceptualisation of food and nutrition education and possible differences between them to ensure a shared understanding of the research phenomenon. Further, the interviewer shared the conceptualisation created for the study but also emphasised that food and nutrition education can be defined in many ways and that there is no single right definition of conceptualisation. For this study, food education was defined as follows, and the interviewer read the concept aloud: ‘Food education in primary schools is practically an interdisciplinary examination of issues, beliefs, and attitudes through discussion, play, example, and experience. It encourages pupils to notice and reflect on food-related phenomena and knowledge from various perspectives. Its objectives can be diverse: they may relate to food culture competence, promoting sustainable lifestyles, understanding food systems, or advancing health. Specifically, when the emphasis is on the health objectives of food education, the term *nutrition education* may be used’.

Interviews were audio-recorded, transcribed verbatim and coded by the first author. The duration of the interviews ranged from 22 to 56 min, with an average duration of 33 min. The transcriptions contained a total of 58 349 Finnish words (an average of 3432 words per interview). The transcripts were uploaded to the qualitative data analysis software Atlas.ti (version 23.4.0.29360) and analysed using theory-driven four-stage content analysis to identify common categories^(33)^. Using this approach, recognised influencing factors were first coded under five deductive themes which were (1) macro-level support, (2) financial implications, (3) core school priorities, (4) developing a common purpose and responsibility and (5) recognition of school and community characteristics^(34)^. The second stage involved recontextualisation, during which the context of the coded texts was revisited, leading to the combination of overlapping codes. In the third stage, the original deductive themes were re-evaluated because of the imbalance of codes between the themes, as themes 1 and 4 contained numerous codes, while theme 2 had very few codes. Therefore, the categories under which the codes were arranged needed to be refined, reorganised and renamed according to the logic of the ecological framework^(35,36)^. Finally, the data were observed through two levels: macro level and school community level (combined physical and social levels). At this third stage, homogenous groups were identified to create categories and sub-categories (i.e. influencing factors).

From the beginning of the analysis through to the end of the third stage, facilitators and challenges directly identified from the data were categorically organised into different groups. In the final fourth stage, the challenges and facilitators were rephrased as opportunities for action, reflecting the researchers’ interpretation. This rephrasing clarified the presentation and interpretation of the results, as challenges often appeared as opposites of the facilitators. Describing them as opportunities provides a clear understanding of the ideal actions that, according to the participants’ perceptions, would be key factors in supporting the implementation of food education in primary schools. The credibility of the findings was established through the presentation of verbatim quotes along with the findings and having regular meetings with the research team during the data analysis stages 3 and 4.

## Results

Headteachers’ and education directors’ perceptions of key factors influencing the implementation of food education in primary schools were categorised into two levels: macro level and school community level. The data revealed that the job descriptions of headteachers and education directors varied slightly. Some worked closely with the everyday life of the school, even teaching classes, while others had offices far from the schools, and their work was primarily administrative, with no contact with pupils. It was also noticeable that the interviewees had slightly different understandings of what food education entails in practice. The understanding of food education practices among headteachers and education directors was focused on activities surrounding school dining and promoting pupils’ wellbeing. However, both groups also included individuals who applied food education in a more holistic manner to examine multidisciplinary phenomena in lessons and integrated it as part of the school culture. These varied perspectives did not reveal a clear pattern in the factors influencing the implementation of food education.

In general, participants rated the following aspects of food education in primary schools on a scale from 1 to 10 (see Table 2; numeral interview questions in phases 2 and 3): their image of food education (M = 7·18), its significance (M = 7·88) and the ease of implementation (M = 7·91). The ‘image’ rating refers to how positively or negatively the term ‘food education’ was perceived (1 = very negative, 10 = very positive). ‘Significance’ refers to how important food education was considered in the context of primary school (1 = not at all significant, 10 = highly significant). ‘Ease of implementation’ reflects how easy or difficult participants found food education to implement in practice (1 = very difficult, 10 = very easy). These evaluations indicated that the interviewees had a positive attitude towards food education, which was influenced by their previous experiences with food education and its active development. The following excerpt reflects one headteacher’s perception of how participation in the project facilitated the ease of implementing food education: ‘Last year, before we were involved in this Tasty School project, it would probably have been around five. But now I say that, of course, it depends on the teacher’s own activity, so it goes much higher, at least between eight and nine for this ease’ (Headteacher 8).

### Macro-level factors influencing the implementation of schools’ food education

This section outlines the key macro-level factors participants identified as shaping the implementation of food education in Finnish primary schools. The research interviews identified four main categories of stakeholders involved in school operations: (1) nationwide initiatives, (2) the Wellbeing Services County, (3) the District Education Department and (4) Food Services (see Table 3). These stakeholders and initiatives operate within broader societal structures that influence how food education is delivered. In Finland, the most influential of these structures is the national, universal school dining system: ‘Well, of course, the school dining is like, um, everyday sort of, uh, an opportunity to implement that food education’ (Education director 5). As a result, food services emerged as one of the key stakeholders:

‘Then I would really consider the role of the kitchen staff important in this matter. Like, if they are willing to invest in things like decorating the dining hall, creating the right atmosphere, cleanliness, or plate models, or whatever could be part of the daily routine, it makes a big difference that the kitchen staff wants our school dining hall to be pleasant. Because not everyone is that actively involved in those things’. (Headteacher 10)

**Table 3.**
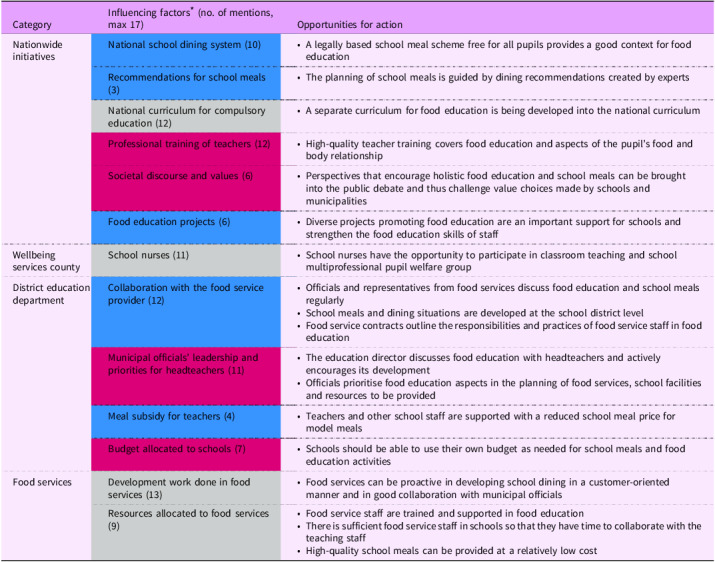
Macro-level opportunities to ideally support the implementation of food education in primary schools according to the participating principals and education directors

* In the ‘influencing factors’ column, the challenging factors – that is, those not yet realised − identified in the data are presented with a red background, the already facilitating factors with a blue background, and those that appeared as both challenging and facilitating factors are presented with a grey background.

Another aspect that stands out in the findings is the importance of multiprofessional collaboration, developing and joint decision-making, particularly between food services and municipal officials:

‘Yeah, maybe it’s more like, when we’ve had those joint events where different schools have been presenting some of the good things they’ve found useful. Then, after discussing them there, and when the kitchen staff supervisors have also been involved, it’s been much easier for another school to adopt those practices afterwards’. (Headteacher 7)

Education directors have the opportunity to prioritise food education and support headteachers in its implementation. However, the majority of headteachers reported that in their municipalities the education director is not involved in the implementation of food education: the headteachers had not received support for implementing food education nor encountered criticism when prioritising it on their own. Education directors reported that, if desired, they could support the implementation of food education by engaging in discussions with headteachers, as they lacked direct contact with teachers. However, nearly none of the directors indicated that they had explicitly taken this action.

The interviewees expressed that the municipality’s strategic papers and curriculum were not currently perceived as highly impactful for the practical implementation of food education. However, their potential future influence was not dismissed. Suggestions were made for how they could be more effective, with an expectation that entries be concrete and provide detailed descriptions of how food education should be implemented.

In Finland, the role of school nurses in primary schools is mainly focused on screenings and individual consultations, rather than health promotion or regular classroom teaching. Their official responsibilities do not include delivering curriculum content, although in some schools they may contribute to specific health-related lessons when invited by teachers. According to the participants, the significance of school nurses’ involvement in food education is typically acknowledged only when individual pupils exhibit problematic eating, at which point the nurse may provide private support or guidance. However, the limited number of school nurses means that opportunities for them to participate actively in daily school life, or in the planning of food education activities, are rare. When school nurses do respond to eating-related concerns, they usually focus on individual health issues rather than whole-class teaching.

### School community-level factors influencing the implementation of schools’ food education

At the school community level, four categories were identified to describe the factors influencing the implementation of food education, as presented in Table 4. The discussions strongly reflected how schools are subject to many expectations and educational responsibilities, with food education being only one of them. This partly explained why food education is not implemented pedagogically, although it was acknowledged that food education takes place in schools every day, at least in connection with school meals. It was emphasised that teachers have too little time for their various educational responsibilities. Therefore, it is the headteacher’s task to guide teachers on what should be deprioritised to ensure sufficient time for food education. Thus, the headteacher’s role is crucial in prioritising what should be implemented.

‘You can influence the attitude as well, and as a headteacher, that is absolutely a significant role when the work plan is being made. So, for example, when we meet and plan together, have meetings for the work plan and such, the headteacher still has quite a significant role in deciding whether this is what we focus on or not. So, I can honestly say that there are sufficient opportunities to influence it’. (Headteacher 7)

‘It is also good that the administration and the headteacher remind that it [food education] exists, like many other things, because primary school education includes so many things, starting from safety to all kinds of topics that aren’t clear subjects, but still need to be taken into account at some point’. (Education director 3)

**Table 4.**
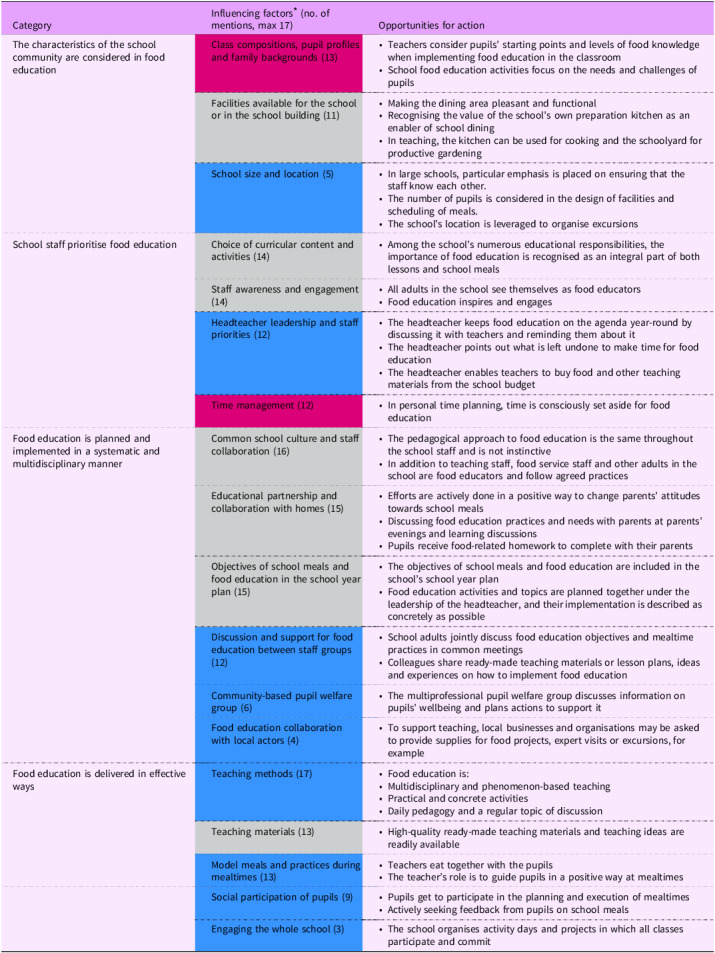
School community-level opportunities to ideally support the implementation of food education in primary schools according to the participating principals and education directors

* In the ‘influencing factors’ column, the challenging factors – that is, those not yet realised – identified in the data are presented with a red background, the already facilitating factors with a blue background, and those that appeared as both challenging and facilitating factors are presented with a grey background.

In the implementation of food education, effective delivery requires careful pedagogical planning. Various aspects of school culture can either support or challenge the achievement of these objectives, and the school food environment may contain influential elements. For example, the presence of a school garden can offer opportunities for hands-on learning, while food environments in the school surroundings, such as a convenience store or other food outlet operating independently of the school, may provide a competing alternative to the school-provided meal and influence pupils’ food choices. The greatest benefit is achieved when the entire school staff is aware of and committed to the objectives and practices of food education.

‘Headteacher 5: In a way, it’s also that there is a culture of activity and an atmosphere in the whole school where food is an important matter and is seen as something significant, so we have sort of common rules there in the dining hall and also regarding the education. Very often, for example, we discuss whether everyone should take milk or not, and whether we eat with a fork and knife, and then when someone doesn’t eat, and so on.

Researcher: Do you discuss this among the teachers or with the pupils?

Headteacher 5: Mmm, both. It’s also that we have common principles and clear guidelines among the adults here’.

## Discussion

This study examined the perceptions of primary school headteachers and education directors regarding the key factors influencing the implementation of food education in Finnish primary schools. The study identified multi-level opportunities to strengthen the implementation, highlighting the systemic nature of the case. In line with earlier studies, the findings indicate that the key factors identified in this research could serve as mechanisms to more firmly embed food education within primary school practices, moving beyond *ad hoc* or time-limited activities^(7)^. Successfully implementing food education through a comprehensive, whole-school and system-wide approach^(37)^ is closely linked to the leadership and prioritisation practices of school headteachers^(38)^, making their role essential in the implementation process.

In Finland, food services refer to the municipally organised provision of free, nutritionally regulated school meals, prepared and served by trained staff and provided equally to all students. While these staff are essential in ensuring the quality and accessibility of school meals, their involvement in formal food education is limited, as pedagogical planning and instruction remain primarily the responsibility of class teachers. In this study, headteachers and education directors viewed food services and food service staff as critical factors in the success of food education, underscoring the central role that daily school meals play in the implementation of food education. This suggests a preference for assigning food education to food services, which risks overlooking the pedagogical planning and expertise required for effective implementation.

At the macro level, the most frequently mentioned factor influencing the implementation of food education was development work done in food services, which referred to the ways in which food services could proactively advance school dining in collaboration with municipal officials. A similar finding was made in a Swedish study^(39)^. The Finnish and Swedish school food services focus strongly on delivering health-promoting meals regulated by national guidelines, but opportunities to link meal provision with pedagogical food education remain underutilised. According to the participants, development efforts typically focus less on ensuring the healthiness of meals, already secured by national recommendations, and more on areas such as sustainability, taste, pupil participation, educators’ pedagogical guidance during mealtimes, and creating a pleasant dining environment and situation. In contrast, elsewhere in the world, the health-promoting quality of food environments in schools is often seen as an area needing improvement and is recognised as an ongoing challenge^(20,25,29)^.

It is noteworthy that many of the challenging and facilitating factors for implementing food education, previously identified in earlier studies and introduced in the introduction, also emerged in the interviews of this study. The contribution of this research is the formulation of opportunities and solutions to overcome challenges and strengthen facilitators. Interestingly, the coverage of teachers’ training regarding food education emerged as a challenge for the implementation of food education, which has also been identified previously^(35)^. At the school community level, teaching methods of food education were widely discussed among the interviewees. The strengths of food education were seen in its multidisciplinary scope, concreteness and offering many hands-on opportunities to pupils, which have earlier been recognised as effective teaching methods^(17,40,41)^.

The greatest barrier to implementing food education globally is the lack of time for teachers (e.g. 20). A lack of time may reflect societal or personal values, as time is allocated to those things that are considered the most important. One solution for time management suggested both in this study and in earlier research is the availability of ready-made lesson plans and teaching materials that support curriculum objectives (e.g. 26). School headteachers could account for these resources in the annual planning process, ensuring they are identified and utilised throughout the school year. Also, the perceived burden of time constraints and workload associated with implementing food education decreases as teachers’ self-efficacy and familiarity grow; professional development that enhances self-efficacy, encourages creative approaches to food education across subjects and strengthens teacher agency is the most effective approach^(35)^.

School culture and educational collaboration between staff groups is a factor that deserves special attention, as the research interviews made it very clear that the headteacher holds a specific responsibility for school culture, and many daily practices are linked to food education within the school. Perhaps the most significant aspect is that food education is planned and implemented in a systematic and multidisciplinary manner, as it seems that learning objectives for food education are often not defined, and therefore the school’s operational culture is not evaluated in light of these objectives^(15,39,42,43)^. It is suggested that the greatest change in school practices is achieved when the headteacher instructs all teachers broadly, rather than focusing on individual support, and does so frequently throughout the school year^(44)^. The findings of this study also indicate that consistently highlighting food education, for example, by asking ‘How’s food education going?’ at the beginning of staff meetings, was perceived as an effective way to support its implementation.

This study preliminarily identified variations in how headteachers and education directors perceived the scope of food education. For some, food education was almost exclusively centred around school meals and dining situations. Others, however, adopted a more holistic perspective, viewing food education as present in all school activities, including classroom lessons. It can be assumed that if food education is predominantly focused on practical dining situations, its pedagogical planning and integration into the curriculum may not occur. The job description and recent experiences with daily school life may also influence this perspective, but verifying these factors and examining them in more detail requires further research.

It is important to acknowledge that headteachers and education directors manage numerous competing priorities within schools, including academic achievement, resource allocation and a range of health and wellbeing initiatives. Food education must therefore be positioned within this complex landscape, supported by clear evidence of its benefits. Research indicates that effective food education can contribute not only to improved nutritional knowledge and eating behaviours but also to enhanced cognitive function and academic performance, reduced health inequalities, long-term public health gains and advances in sustainability and planetary health^(6,7,17,18,37,45–51)^. By framing food education as integral to these broader educational and societal objectives, policymakers and school leaders may be more inclined to prioritise its implementation and integration into school practices. Such alignment strengthens the case for sustained leadership engagement and resource commitment, ensuring that food education is not perceived as a peripheral activity but as a core component of holistic pupil development.

There is relatively little qualitative research on primary school headteachers’ and education directors’ perceptions of the implementation of food education. In the study, the interviewed headteachers and education directors were from different regions of Finland, with a particular focus on Eastern and Southern Finland, which contributes to the reliability of the results. Except for three headteachers and one education director, all those who received the research invitation participated in the study, so the research data effectively reflects the selected target group. The validity of the results is strengthened by the fact that the headteachers, in particular, who participated in the study had previously been involved with the Tasty School project with their schools, providing them with recent experiences in food education. This ensured that the interviews could engage in comprehensive discussions about food education and that the interviewees had direct experiences with factors that either challenge or facilitate its implementation.

The limitations of the study should be considered. Since this study was connected to the Tasty School project, participants may have already had a particular interest in food education and, consequently, might not fully represent the general perceptions of school leaders. Additionally, school systems vary, and while comparisons across different countries require adjustments, the results can still be utilised where applicable. Despite these limitations, the findings are particularly useful in countries, municipalities and schools that provide free school meals to all pupils through food services.

### Conclusions

The findings suggest that successful implementation of food education in primary schools necessitates a school culture that prioritises food education. This includes setting teaching and learning objectives in the curriculum, allocating sufficient time for pedagogical planning, ensuring access to resources for purchasing food items and creating supportive learning environments. School headteachers play a central role in promoting the development of such a culture. The study highlights important findings to support the implementation of food education at both school and municipal levels. The results emphasise the important role of headteachers, but the issues raised also require the willingness of municipal officials and policymakers to promote them.

## Supporting information

Laitinen et al. supplementary material Laitinen et al. supplementary material

## Supplementary material

For supplementary material accompanying this paper visit https://doi.org/10.1017/S1368980025101547
